# Corticosteroids for Dengue – Why Don't They Work?

**DOI:** 10.1371/journal.pntd.0002592

**Published:** 2013-12-12

**Authors:** Thi Hanh Tien Nguyen, Than Ha Quyen Nguyen, Tuan Trung Vu, Jeremy Farrar, Truong Long Hoang, Thi Hoai Tam Dong, Van Ngoc Tran, Khanh Lam Phung, Marcel Wolbers, Stephen S. Whitehead, Martin L. Hibberd, Bridget Wills, Cameron P. Simmons

**Affiliations:** 1 Oxford University Clinical Research Unit, Hospital for Tropical Diseases, Ho Chi Minh City, Viet Nam; 2 Centre for Tropical Medicine, Nuffield Department of Medicine, University of Oxford, Oxford, United Kingdom; 3 Genome Institute of Singapore, Singapore; 4 Hospital for Tropical Diseases, Ho Chi Minh City, Viet Nam; 5 Laboratory of Infectious Diseases (LID), National Institutes of Allergy and Infectious Diseases, Bethesda, Maryland, United States of America; 6 Nossal Institute of Global Health, School of Population and Global Health, University of Melbourne, Parkville, Victoria, Australia; Pediatric Dengue Vaccine Initiative, United States of America

## Abstract

**Background:**

Dysregulated immune responses may contribute to the clinical complications that occur in some patients with dengue.

**Findings:**

In Vietnamese pediatric dengue cases randomized to early prednisolone therapy, 81 gene-transcripts (0.2% of the 47,231 evaluated) were differentially abundant in whole-blood between high-dose (2 mg/kg) prednisolone and placebo-treated patients two days after commencing therapy. Prominent among the 81 transcripts were those associated with T and NK cell cytolytic functions. Additionally, prednisolone therapy was not associated with changes in plasma cytokine levels.

**Conclusion:**

The inability of prednisolone treatment to markedly attenuate the host immune response is instructive for planning future therapeutic strategies for dengue.

## Author Summary

Dengue is an acute, mosquito-borne febrile illness and around 390 million cases occur annually in more than 100 countries. A host pro-inflammatory immune response is widely believed to contribute to the clinical complications that occur in some patients with dengue. Synthetic glucocorticoids, which are immunomodulatory agents commonly used in medicine, have been suggested as a therapy for dengue. We recently performed a randomized, controlled trial of early oral glucocorticoid therapy in 225 dengue cases in Vietnam, comparing a three day regimen of high (2 mg/kg) or low (0.5 mg/kg) dose prednisolone with placebo. Here, we report on immunological changes occurring during prednisolone therapy with a view to understanding the lack of clinical benefit by glucocorticoid therapy and to guide future intervention strategies for dengue. In whole-blood gene expression arrays we found 81 transcripts from 64 genes differentially abundant between high-dose prednisolone and placebo treated patients. Prominent were the genes associated with T and NK cell cytolytic functions. These results are a reminder that the mechanisms causally behind some of the complications of dengue (e.g. altered capillary permeability) are very poorly understood and represent a major knowledge gap in our understanding of disease pathogenesis that also undermines attempts to improve clinical management.

## Introduction

Dengue is an acute, mosquito-borne illness caused by any of the four types of dengue virus (DENV1-4). There are an estimated 390 million symptomatic and asymptomatic infections per year [1]. The clinical evolution is variable, ranging from non-specific febrile illness to severe and sometimes fatal disease. One of the commonest complications observed is a transient vasculopathy, manifesting as increased vascular permeability with altered haemostasis, typically 3–6 days after fever onset. Dysregulated host immune responses, particularly those associated with secondary infections, are widely held to contribute mechanistically to the vasculopathy that characterizes severe dengue [2]. No specific therapies or licensed vaccines are currently available and management relies on assiduous supportive care.

Synthetic glucocorticoids are frequently employed as adjunctive therapy in disease states where the host immune response is thought to be a significant contributor to disease pathogenesis. We recently performed a randomized, controlled trial of early oral prednisolone therapy in 225 confirmed pediatric dengue cases [3]. Although the trial was primarily designed to assess safety we were unable to detect any reduction in the severity of plasma leakage or other recognised complications of dengue. We report here on immunological correlates of prednisolone therapy in this trial with a view to understanding the lack of clinical benefit imparted by prednisolone and to guide future intervention strategies for dengue.

## Materials and Methods

### Patient population and clinical methods

A randomized, placebo-controlled double-blind trial assessing the safety of early oral corticosteroid therapy in dengue patients was conducted at the Hospital for Tropical Diseases, Ho Chi Minh City, Vietnam between August 2009 and January 2011 as approval from the Ethical Committee of the Ministry of Health of Vietnam (2407/QĐ-BYT) and the Oxford Tropical Research Ethics Committee (OxTREC 33-08) and has been reported elsewhere [3]. The trial registration number is ISRCTN39575233. Briefly, patients aged from 5–20 years with fever for less than 72 hrs and a positive dengue NS1 rapid test were randomly allocated to oral treatment with high-dose prednisolone (2 mg/kg), low-dose prednisolone (0.5 mg/kg) or identical placebo for 3 days provided the patient or their parent/guardian gave written informed consent and children 12–17 years gave assent; all patients recovered fully and we found no association between treatment allocation and any of the predefined clinical, haematological or virological endpoints. The research blood specimens that form the basis of the work described here were collected as part of the trial protocol at pre-specified time-points: enrolment (pre-treatment); 2 days post initiation of treatment; and at follow-up in late convalescence (median 29 (IQR 27, 30) days after enrolment). To facilitate interpretation of the findings with respect to defervescence, the first day that the temperature fell to 37.5°C or less and remained below this level for 48 hours or until discharge was taken as the day of deferescence; fever day 0 was defined as the calendar day of defervescence, with days before this point numbered consecutively as fever days −1, − 2, − 3 respectively.

### Cytokines

Eleven cytokines (IL-1β, IL-2, IL-4, IL-5, IL-6, IL-10, IL-12p70, IL-13, IFNγ, TNFα and IP10) were quantified using a multiplex biometric immunoassay following the instructions of the manufacturer (Bio-Plex Precision Pro Assays, Human cytokine 10-Plex, Bio-Rad Inc., USA). The limits of detection were as follows: 0.23 (IL-1β), 0.84 (IL-2), 0.14 (IL-4), 1.5 (IL-5), 1.23 (IL-6), 0.96 (IL-10), 0.2 (IL-12p70), 1.19 (IL-13), 0.34 (IFN-γ), 0.14 (TNF-α) and 10 (IP10), all pg/ml.

### Gene expression microarray and PCR validation

The gene expression microarray assay, and the procedures for normalization and analysis of the microarray data were conducted as described elsewhere [4]. Samples from the first 123 consecutive patients enrolled in the study were used as the “discovery” cohort.

A fluidigm system (Fluidigm Corp., USA) was used for realtime PCR (RT-PCR) validation of those differentially abundant transcripts identified in the gene expression microarray, using samples from the whole patient cohort and following the manufacturer's instructions. The delta Ct value for each gene was calculated by subtracting the Ct value of the gene of interest from the Ct value of 18S, the house-keeping gene.

### Data analysis

All group comparisons of microarray data were based on ANOVA with correction for multiple testing with the Benjamini-Hochberg method, as implemented in the GeneSpring Software (Silicon Genetics). A fold change of 1.5 was defined as the cut-off for screening significant entities. We used multivariable linear regression modeling for all comparisons of PCR results, expressed as delta Ct values, between the treatment arms. For the overall comparison across treatment arms, a linear trend test was used. In view of the likely evolution of gene expression during the illness episode, and the known associations of many of the genes of interest to immune parameters, we adjusted for day of illness and the absolute neutrophil and lymphocyte counts. Additionally we adjusted for the pre-treatment value when examining within-patient changes in delta Ct over time. P values for testing of multiple PCR results were corrected using the Benjamini-Hochberg method. The relative expression ratios of the genes between the treatment arms were estimated using the delta delta Ct formula: R = 2^−ΔΔCt^ with corresponding 95% confidence intervals based on the bootstrap. Genes with significantly different relative expression ratios between treatment arms (i.e. adjusted p<0.05 and 95%CI not including 1) were considered up or down regulated as appropriate.

Multivariable linear regression was used for all comparisons of log-transformed values of cytokine concentrations adjusting for day of illness and pre-treatment values and linear trend tests performed as described above. All the analyses were corrected for multiple tests using the Benjamini-Hochberg method. All analyses other than those pertaining to the microarray data were performed using R, version R2.13.2 (R Foundation for Statistical Computing, Vienna, Austria).

#### Microarray data accession number

The GEO accession number for microarray data is GSE40165

## Results

### Differentially abundant gene expression linked to high-dose prednisolone therapy

**Figure 1:**
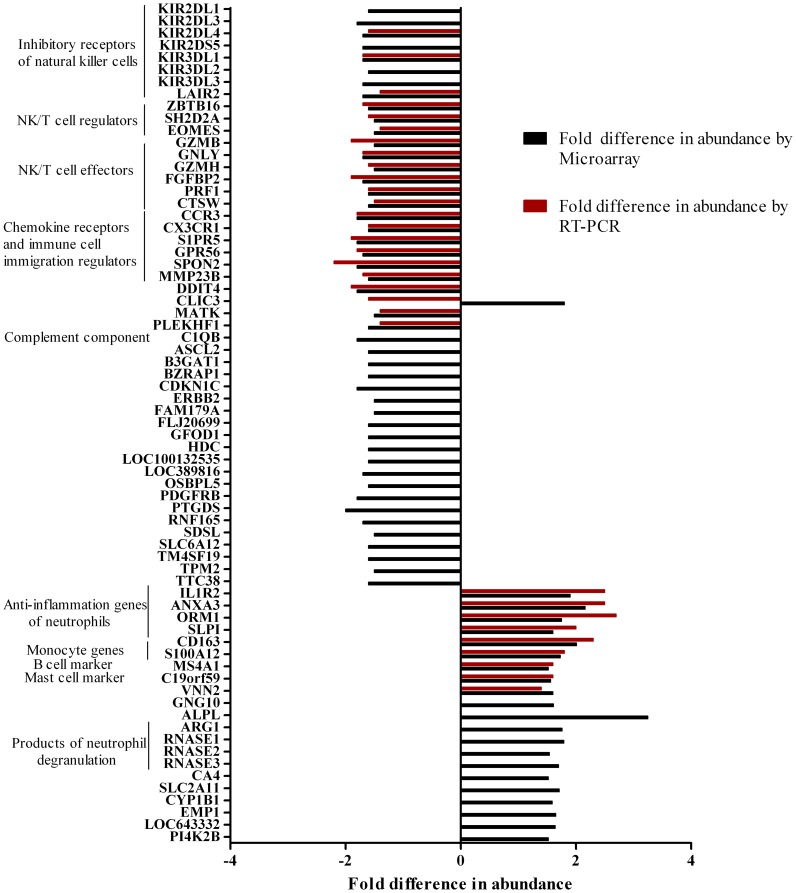
Fold difference in transcript abundance between high-dose prednisolone and placebo treated patients 2 days post initiation of treatment. Shown is the ratio in abundance of transcripts measured by microarray and by RT-PCR for the 67 genes that distinguished high-dose prednisolone treated patients from placebo treated patients. Where results from multiple probes in the microarray were available for 1 gene, we used the mean result. Genes have been grouped and annotated according to their recognized biological functions. Median (IQR) times from a) the start of treatment and b) the most recent dose, to the time of the microarray sample were similar; 43 (42–43) hours and 23 (22–24) hours for the high-dose prednisolone recipients, and 43 (42–44) hours and 22 (22–23) hours for the placebo recipients.

Baseline characteristics for the first 123 dengue patients consecutively enrolled in the trial who formed the discovery cohort are described in Table 1 together with similar information for the 223 patients used for the PCR validation; clinical and laboratory features were similar across the three intervention arms. At enrolment, none of the 47,231 transcripts evaluated in the microarray were differentially abundant between groups of patients allocated to different treatment arms. Similarly, there were no transcripts differentially abundant between treatment groups in late convalescence, or between placebo and low-dose prednisolone patients 2 days after commencing treatment. By contrast, 81 differentially abundant transcripts (25 that were more abundant and 56 less abundant), representing 67 genes, were detected when comparing whole-blood gene expression profiles between the high-dose prednisolone and placebo groups 2 days after commencing treatment. RT-PCR validation measurements were targeted to a subgroup of 31 (46%) of the 67 genes on the basis of their having plausible roles in immune function. RT-PCR validation was performed with 600 whole blood RNA samples collected at baseline (N = 208), 2 days after starting treatment (N = 200), and in late-convalescence (N = 192) from 223 (99%) of the 225 patients in the trial. With the exception of one target (CLIC3), RT-PCR measurements were entirely concordant with the microarray findings (Figure 1). Annotation of RT-PCR validated gene elements enabled functional grouping according to their recognized roles in NK and T cell cytolytic function, T cell activation and innate immune responses (Figure 1). These analyses were extended by investigating a prednisolone dose-response (placebo, low-dose, high dose) relationship. After adjustment for the a priori defined variables (day of illness, absolute neutrophil and lymphocyte counts, baseline transcript abundance), a highly significant prednisolone dose-related effect on gene transcript abundance was observed for all 31 transcripts (Table S1). Collectively, these results define a discrete gene transcript profile that is associated with high-dose prednisolone therapy in dengue patients.

**Table 1:** Enrolment characteristics of patients sampled for the microarray and RT-PCR investigations.

	Microarray population			RT-PCR population		
	(N = 123(a))			(N = 223(b))		
	Placebo	Low-dose	High-dose	Placebo	Low-dose	High-dose
	(N = 40)	(N = 42)	(N = 41)	(N = 75)	(N = 74)	(N = 74)
**Sex, female**	11 (28)	11 (26)	16 (39)	19 (25)	21 (28)	24 (32)
**Age, years**	13 (11–14)	11 (10–14)	12 (10–13)	13 (12–15)	12 (11–14)	12 (10–14)
**Day of illness**						
**Day 1**	0 (-)	0 (-)	1 (2)	0 (-)	1 (1)	1 (1)
**Day 2**	17 (42)	14 (33)	16 (39)	28 (37)	23 (31)	29 (39)
**Day 3**	23 (58)	28 (67)	24 (59)	47 (63)	50 (68)	44 (59)
**Fever day** *	−5 (−6, −4)	−4 (−6, −4)	−5 (−6, −4)	−5 (−6, −4)	−4 (−6, −4)	−4 (−6, −4)
**Serotype**						
**DENV-1**	27 (68)	27 (64)	29 (71)	41 (55)	46 (62)	49 (66)
**DENV-2**	11 (28)	8 (19)	3 (7)	29 (39)	17 (23)	11 (15)
**DENV-3**	2 (5)	5 (12)	6 (15)	4 (5)	9 (12)	10 (14)
**DENV-4**	0 (-)	2 (5)	3 (7)	1 (1)	2 (3)	4 (5)
**Total WBC (10^3^/µL)**	3.1 (2.5–4.3)	4.0 (3.0–5.9)	4.2 (2.7–5.8)	3.8 (2.7–5.0)	3.7(c) (3.0–5.4)	4.2(c) (3.0–5.2)
**Neutrophils (10^3^/µL)**	2.2 (1.3–3.1)	2.7 (1.8–4.1)	2.8 (2.0–3.6)	2.6 (1.7–3.5)	2.5(c) (1.7–3.9)	2.9(c) (2.0–3.8)
**Lymphocytes (10^3^/µL)**	0.7 (0.5–0.9)	0.7 (0.5–1.0)	0.7 (0.5–1.0)	0.7 (0.6–0.9)	0.7(c) (0.5–0.9)	0.7(c) (0.5–1.0)

Continuous variables are summarized as median (IQR), categorical variables are summarized as number and frequency (%).

(a) 358 whole-blood RNA samples from the first 123 consecutively enrolled patients were included in the microarray experiment. 11 follow-up samples were missing.

(b) 600 samples from 223 patients (including the123 patients that were sampled for the microarray) were included in the RT-PCR validation study. (15 pre-treatment, 23 post-treatment and 31 follow-up samples were either missing or of poor quality and were therefore not included).

(c) There was one missing value.

(*) Fever day (median (IQR) refers to the day of enrolment relative to the day of defervescence, defined as fever day 0.

### Plasma cytokines and prednisolone therapy

Concentrations of 11 cytokines and chemokines were measured in 636 serial plasma samples from 222 patients at the three time-points. Although cytokine/chemokine concentrations were within the detectable range in 98% of samples tested, their levels were not significantly elevated during the acute phase of illness compared to follow-up, and there were no significant differences between treatment groups 2 days after starting therapy (Table 2).

**Table 2:** Plasma cytokine concentrations 2 days post initiation of treatment.

Cytokine	Plasma concentration 2 days after starting therapy			Trend test**	
	High-dose prednisolone	Low-dose prednisolone	Placebo	Multiplicative effect	Adjusted P
	(Fever day* = −2 (−4, −2))	(Fever day = −2 (−4, −2))	(Fever day = −3 (−4, −2))	(95%CI)	
IL-1β	11.3	12.5	13.1	0.90	0.24
	(8.5–15.1)	(10.7–16.3)	(10.2–18.3)	(0.75–1.07)	
IL-2	50.6	54.6	50.9	0.89	0.31
	(37.6–66.6)	(40.2–69.5)	(38.4–74.6)	(0.72–1.11)	
IL-4	13.4	14.1	13.3	0.91	0.29
	(10.2–17.2)	(10.2–16.9)	(10.0–19.0)	(0.77–1.08)	
IL-5	79	79	79.3	0.91	0.42
	(62.6–101.1)	(63.3–92.0)	(64.6–96.4)	(0.73–1.14)	
IL-6	132.9	125.7	140.1	0.98	0.83
	(93.3–183)	(106.4–162.4)	(102.3–199.6)	(0.80–1.20)	
IL-10	119.4	118.2	126.7	0.98	0.65
	(80.8–166)	(96.7–152.6)	(98.4–167.4)	(0.92–1.05)	
IL-12p70	24	23.1	23.2	0.97	0.70
	(16.0–32.4)	(16.0–30.1)	(15.8–30.2)	(0.82–1.14)	
IL-13	26.4	23.6	25.6	0.94	0.42
	(17.3–40.1)	(17.1–40.1)	(18.5–36.1)	(0.80–1.10)	
IFN-γ	56.5	58.1	62.2	0.97	0.48
	(41.0–96.0)	(42.4–88.5)	(41.8–85.3)	(0.87–1.06)	
TNFα	13.2	12.7	14.5	0.95	0.56
	(10.6–17.6)	(10.2–16.2)	(10.0–18.1)	(0.780–1.13)	
IP-10	8398	8097.5	8099	1.06	0.14
	(5,099.2–11,506.5)	(4,998.9–11,424.4)	(4,701.8–10,630.3)	(0.98–1.14)	

All data are presented as median (IQR) values.

* Fever day (median (IQR) refers to the day of sampling relative to the day of defervescence, defined as fever day 0.

** Trend test using multivariable linear regression of log transformed values adjusted for pre-treatment value and day of illness at enrolment. The effect corresponds to the estimated multiplicative difference between low-dose vs. placebo or high-dose vs. low-dose, respectively, which were assumed to be identical as a linear dose-response effect was estimated.

## Discussion

The current study was linked to a randomized controlled trial of early prednisolone therapy for dengue that demonstrated the safety of prednisolone but did not provide evidence of improved clinical or laboratory outcomes for patients [3]. Here we provide insights into these trial findings by identifying a surprisingly small prednisolone-associated footprint (just 81 transcripts differentially abundant from 47,231 evaluated) on the whole-blood gene expression profile that manifests during DENV infection. Furthermore, acute-phase plasma cytokine concentrations were not measurably attenuated by prednisolone treatment. The limited immunomodulation achieved by prednisolone is consistent with it having negligible measurable benefits in the clinical trial in which this current study was nested.

Dysregulated immune responses are widely believed to contribute to the pathogenesis of severe dengue [2], and hence corticosteroid therapy has been trialed in several small studies of patients with shock due to vascular leakage [5], [6]. No benefit was demonstrated in those studies, but treatment was initiated when shock was already established, when it is probably too late to modulate the host immune response. In our recent intervention study prednisolone was commenced during the early febrile phase, but we were still unable to demonstrate any amelioration in the severity of plasma leakage. Against a backdrop where the immunomodulatory actions of corticosteroids are well established [7], [8], it is surprising that we did not observe stronger signals of immune-modulation. The absence of a measurable impact of prednisolone on plasma cytokine concentrations two days after enrolment reflects the absence of any significant elevation of the 11 cytokines/chemokines in the acute phase compared to convalescence. This is at odds with a body of literature indicating elevated plasma/serum cytokine concentrations are a prominent feature in the host response [9]. However a limitation of this current study, unlike previous work [10], [11], was that we did not measure cytokine/chemokine concentrations in serial daily plasma specimens and therefore we may have missed transient changes in particular markers.

Changes in the whole-blood host gene expression profile occurred in patients that had received 2 mg/kg prednisolone (but not 0.5 mg/kg) compared to placebo-treated patients. This is to our knowledge the first ex vivo and “global’ investigation of the effect of prednisolone on the host immune response during an infectious disease. A striking finding, that was independent of the blood lymphocyte count, was the prednisolone-associated under-abundance of transcripts representing granzyme B (GZMB), granzyme H (GZMH), granulysin (GNLY), perforin (PRF1), Ksp37 (FGFBP2) and cathepsin W (CTSW), each of which is associated with the secretory and cytolytic activities of T and NK cells. Taken together, the diminished abundance of transcripts encoding T and NK effector proteins might suggest impaired anti-viral cytolytic responses during high-dose prednisolone therapy. However prolonged viremia levels were not observed in prednisolone-treated patients [3]. It is plausible that the lower transcript abundance of these elements in whole blood is not biologically significant in terms of resolving infection and that other components of the immune response, such as antibodies or complement, are more important and/or compensatory.

Transcripts from neutrophils, independent of the absolute neutrophil count, were more abundant in high-dose prednisolone treated patients. This included three neutrophil markers (IL1R2, S100A12 and ORM1), all known to be corticosteroid induced [12]–[14]; interestingly these markers were also independently identified as being elevated at a similar timepoint in relation to defervescence (fever day −2 to −3) in a previous study of 35 Vietnamese children who subsequently developed DSS ([4]). Further studies will be required to understand if prednisolone exacerbates the functional neutrophil response in addition to its well-described demargination effects [15] [16].

There are limitations to our study. We did not measure complement activation yet there is good evidence that complement is consumed and split products generated in the course of dengue and that this might be important to pathogenesis [17]. Our sampling schedule may have missed transient but important differences in cellular gene expression signals between patients in different treatment arms. Prednisolone may also have actions in tissues that are not revealed in whole blood. Nevertheless the results of this study, coupled with the findings from the clinical trial [3], suggest that early prednisolone therapy has little impact on the host immune response or the clinical evolution of dengue. One possible explanation is that early prednisolone therapy is “too little, too late” to attenuate the infection-driven processes that lead to the altered capillary permeability, thrombocytopenia, and haemostatic derangements. We can only speculate that even earlier treatment, or higher dose therapy, might have led to a greater prednisolone impact on the immune response and clinical/laboratory phenotype. Notwithstanding these limitations, the results described here underscore the challenge of modulating an immune response that has been driven by days of DENV replication in host tissues. More fundamentally, these results are a reminder that although immune-driven pathophysiological changes are good candidates to explain capillary permeability, the precise causal mechanisms remain poorly understood. This represents a major knowledge gap in our understanding of disease pathogenesis that also undermines development of specific therapies.

## Supporting Information

Table S1 **Genes with significantly different transcript abundance between patients treated with high-dose prednisolone and placebo.** * Trend test using multivariable linear regression of log transformed values adjusted for pre-treatment value and day of illness at enrolment. The effect corresponds to the estimated multiplicative difference between low-dose vs. placebo or high-dose vs. low-dose, respectively, which were assumed to be identical as a linear dose-response effect was estimated. **^a^** All p values were corrected with Benjamini-Hochberg method for multiple test correction. **NS** denotes genes that were not significantly different in gene expression between the patients receiving low dose prednisolone and placebo and/or that the 95% confidence interval of the relative expression ratio contained 1.
(DOC)

## References

[1] Bhatt S, Gething PW, Brady OJ, Messina JP, Farlow AW, et al (2013) The global distribution and burden of dengue. Nature 496: 504–507. 23563266 10.1038/nature12060 PMC3651993

[2] Rothman AL (2011) Immunity to dengue virus: a tale of original antigenic sin and tropical cytokine storms. Nat Rev Immunol 11: 532–543. 21760609 10.1038/nri3014

[3] Tam DT, Ngoc TV, Tien NT, Kieu NT, Thuy TT, et al (2012) Effects of short-course oral corticosteroid therapy in early dengue infection in Vietnamese patients: a randomized, placebo-controlled trial. Clin Infect Dis 55: 1216–1224. 22865871 10.1093/cid/cis655 PMC3466094

[4] Hoang LT, Lynn DJ, Henn M, Birren BW, Lennon NJ, et al (2010) The early whole-blood transcriptional signature of dengue virus and features associated with progression to dengue shock syndrome in Vietnamese children and young adults. J Virol 84: 12982–12994. 20943967 10.1128/JVI.01224-10 PMC3004338

[5] Sumarmo, Talogo W, Asrin A, Isnuhandojo B, Sahudi A (1982) Failure of hydrocortisone to affect outcome in dengue shock syndrome. Pediatrics 69: 45–49. 7054760

[6] Tassniyom S, Vasanawathana S, Chirawatkul A, Rojanasuphot S (1993) Failure of high-dose methylprednisolone in established dengue shock syndrome: a placebo-controlled, double-blind study. Pediatrics 92: 111–115. 8516054

[7] Flammer JR, Dobrovolna J, Kennedy MA, Chinenov Y, Glass CK, et al (2010) The type I interferon signaling pathway is a target for glucocorticoid inhibition. Mol Cell Biol 30: 4564–4574. 20679482 10.1128/MCB.00146-10 PMC2950533

[8] Singh Nikhil JR, Jane Tucker M (2004) Mechanisms of glucocorticoid-mediated antiinflammatory and immunosuppressive action. Paediatric and Perinatal Drug Therapy 6: 107–115.

[9] Nguyen TH, Lei HY, Nguyen TL, Lin YS, Huang KJ, et al (2004) Dengue hemorrhagic fever in infants: a study of clinical and cytokine profiles. J Infect Dis 189: 221–232. 14722886 10.1086/380762

[10] Green S, Vaughn DW, Kalayanarooj S, Nimmannitya S, Suntayakorn S, et al (1999) Early immune activation in acute dengue illness is related to development of plasma leakage and disease severity. J Infect Dis 179: 755–762. 10068569 10.1086/314680

[11] Libraty DH, Endy TP, Houng HS, Green S, Kalayanarooj S, et al (2002) Differing influences of virus burden and immune activation on disease severity in secondary dengue-3 virus infections. J Infect Dis 185: 1213–1221. 12001037 10.1086/340365

[12] Baumann H, Prowse KR, Marinkovic S, Won KA, Jahreis GP (1989) Stimulation of hepatic acute phase response by cytokines and glucocorticoids. Ann N Y Acad Sci 557: 280–295 discussion 295-286. 2472090 10.1111/j.1749-6632.1989.tb24021.x

[13] Buechler C, Ritter M, Orso E, Langmann T, Klucken J, et al (2000) Regulation of scavenger receptor CD163 expression in human monocytes and macrophages by pro- and antiinflammatory stimuli. J Leukoc Biol 67: 97–103. 10648003

[14] Muller B, Peri G, Doni A, Perruchoud AP, Landmann R, et al (2002) High circulating levels of the IL-1 type II decoy receptor in critically ill patients with sepsis: association of high decoy receptor levels with glucocorticoid administration. J Leukoc Biol 72: 643–649. 12377932

[15] Nakagawa M, Terashima T, D'Yachkova Y, Bondy GP, Hogg JC, et al (1998) Glucocorticoid-induced granulocytosis: contribution of marrow release and demargination of intravascular granulocytes. Circulation 98: 2307–2313. 9826319 10.1161/01.cir.98.21.2307

[16] Liu L, Wang YX, Zhou J, Long F, Sun HW, et al (2005) Rapid non-genomic inhibitory effects of glucocorticoids on human neutrophil degranulation. Inflamm Res 54: 37–41. 15723203 10.1007/s00011-004-1320-y

[17] Avirutnan P, Punyadee N, Noisakran S, Komoltri C, Thiemmeca S, et al (2006) Vascular leakage in severe dengue virus infections: a potential role for the nonstructural viral protein NS1 and complement. J Infect Dis 193: 1078–1088. 16544248 10.1086/500949

