# Supplementary material for: Corticosteroids for Dengue – Why Don't They Work?
Source: PLoS Negl Trop Dis. 2013 Dec 12;7(12):e2592. doi: 10.1371/journal.pntd.0002592 (PMC3861232; doi:10.1371/journal.pntd.0002592)
Supplement: Table S1 — Genes with significantly different transcript abundance between patients treated with high-dose prednisolone and placebo. * Trend test using multivariable linear regression of log transformed values adjusted for pre-treatment value and day of illness at enrolment. The effect corresponds to the estimated multiplicative difference between low-dose vs. placebo or high-dose vs. low-dose, respectively, which were assumed to be identical as a linear dose-response effect was estimated. a All p values were corrected with Benjamini-Hochberg method for multiple test correction. NS denotes genes that were not significantly different in gene expression between the patients receiving low dose prednisolone and placebo and/or that the 95% confidence interval of the relative expression ratio contained 1. (DOC) [file pntd.0002592.s001.doc]

- **Table S1**: Genes with significantly different transcript abundance between patients treated with high-dose prednisolone and placebo

| - **Gene Symbol** | - **Microarray** | | - **RT-PCR** | | |
| --- | --- | --- | --- | --- | --- |
|  | - **Abundance** | - **Fold change** | **Absolute relative expression ratio** | - **Absolute relative expression ratio** | - **Trend test^*^,** - **P value^a^** |
|  |  |  | **(95% CI)** | - **(95% CI)** |  |
|  |  |  | **High-dose vs Placebo** | - **Low-dose vs Placebo** |  |
| - ORM1 | - Higher | - 1.8 | - 2.7 (1.5 - 3.5) | - NS | - 1.20E-06 |
| - ANXA3 | - Higher | - 2.2 | - 2.5 (1.7 - 3.1) | - 1.4 (1.0 - 1.8) | - 5.01E-08 |
| - IL1R2 | - Higher | - 1.9 | - 2.5 (1.5 - 3.1) | - 1.7 (1.0 - 2.1) | - 1.40E-07 |
| - CD163 | - Higher | - 2.0 | - 2.3 (1.7 - 2.8) | - 1.5 (1.1 - 1.8) | - 2.77E-08 |
| - SLPI | - Higher | - 1.6 | - 2.0 (1.3 - 2.6) | - NS | - 1.04E-04 |
| - S100A12 | - Higher | - 1.7 | - 1.8 (1.1 - 2.2) | - NS | - 6.54E-06 |
| - MS4A1 | - Higher | - 1.5 | - 1.6 (1.2 - 1.8) | - 1.2 (1.0 - 1.4) | - 1.31E-05 |
| - C19orf59 | - Higher | - 1.6 | - 1.6 (1.1 - 1.9) | - NS | - 9.64E-04 |
| - VNN2 | - Higher | - 1.6 | - 1.4 (1.1 - 1.7) | - NS | - 2.78E-02 |
| - KIR3DL1 | - Lower | - 1.7 | - 1.7 (1.4 - 2.6) | - 1.4 (1.1 - 2.1) | - 1.01E-03 |
| - KIR2DL4 | - Lower | - 1.7 | - 1.6 (1.3 - 2.2) | - 1.3 (1.1 - 1.8) | - 3.88E-05 |
| - LAIR2 | - Lower | - 1.7 | - 1.4 (1.2 - 1.8) | - 1.3 (1.1 - 1.6) | - 2.46E-03 |
| - ZBTB16 | - Lower | - 1.6 | - 1.7 (1.4 - 2.2) | - 1.5 (1.3 - 1.9) | - 6.42E-07 |
| - SH2D2A | - Lower | - 1.5 | - 1.6 (1.3 - 2.0) | - 1.4 (1.2 - 1.8) | - 5.09E-04 |
| - EOMES | - Lower | - 1.5 | - 1.4 (1.2 - 1.9) | - 1.4 (1.2 - 1.7) | - 8.20E-03 |
| - GZMB | - Lower | - 1.5 | - 1.9 (1.5 - 2.6) | - 1.5 (1.2 - 2.0) | - 1.57E-04 |
| - GNLY | - Lower | - 1.7 | - 1.7 (1.4 - 2.2) | - 1.3 (1.1 - 1.7) | - 3.67E-04 |
| - GZMH | - Lower | - 1.5 | - 1.6 (1.3 - 2.2) | - 1.3 (1.1 - 1.8) | - 9.01E-04 |
| - PRF1 | - Lower | - 1.6 | - 1.6 (1.3 - 2.2) | - 1.4 (1.2 - 1.9) | - 1.17E-03 |
| - CTSW | - Lower | - 1.6 | - 1.5 (1.2 - 1.9) | - NS | - 1.26E-02 |
| - CCR3 | - Lower | - 1.8 | - 1.8 (1.4 - 2.5) | - 1.4 (1.2 - 2.0) | - 5.63E-08 |
| - CX3CR1 | - Lower | - 1.6 | - 1.6 (1.3 - 2.1) | - 1.3 (1.1 - 1.7) | - 1.16E-03 |
| - S1PR5 | - Lower | - 1.8 | - 1.9 (1.6 - 2.5) | - 1.5 (1.2 - 1.9) | - 9.89E-06 |
| - GPR56 | - Lower | - 1.7 | - 1.8 (1.5 - 2.6) | - 1.4 (1.1 - 2.0) | - 4.74E-04 |
| - SPON2 | - Lower | - 1.8 | - 2.2 (1.9 - 3.0) | - 1.6 (1.3 - 2.2) | - 2.04E-07 |
| - DDIT4 | - Lower | - 1.8 | - 1.9 (1.7 - 2.4) | - 1.5 (1.3 - 1.9) | - 5.67E-09 |
| - FGFBP2 | - Lower | - 1.7 | - 1.9 (1.5 - 2.7) | - 1.6 (1.3 - 2.2) | - 2.06E-04 |
| - MMP23B | - Lower | - 1.6 | - 1.7 (1.4 - 2.4) | - 1.4 (1.2 - 1.9) | - 1.01E-03 |
| - CLIC3 | - Higher | - 1.8 | - 1.6 (1.3 - 2.1) | - 1.3 (1.1 - 1.7) | - 3.55E-04 |
| - MATK | - Lower | - 1.5 | - 1.4 (1.2 - 1.8) | - NS | - 7.13E-03 |
| - PLEKHF1 | - Lower | - 1.6 | - 1.4 (1.2 - 1.8) | - NS | - 6.41E-03 |

- * Trend test using multivariable linear regression of log transformed values adjusted for pre-treatment value and day of illness at enrolment. The effect corresponds to the estimated multiplicative difference between low-dose vs. placebo or high-dose vs. low-dose, respectively, which were assumed to be identical as a linear dose-response effect was estimated.

**^a^** All p values were corrected with Benjamini-Hochberg method for multiple test correction.

- **NS** denotes genes that were not significantly different in gene expression between the patients receiving low dose prednisolone and placebo and/or that the 95% confidence interval of the relative expression ratio contained 1.
